# Horizon quantum mechanics for coherent quantum black holes

**DOI:** 10.1140/epjp/s13360-025-06065-x

**Published:** 2025-02-20

**Authors:** Wenbin Feng, Andrea Giusti, Roberto Casadio

**Affiliations:** 1 Dipartimento di Fisica e Astronomia, Università di Bologna, via Irnerio 46, 40126 Bologna, Italy; 2 I.N.F.N., Sezione di Bologna, I.S. FLAG, viale B. Pichat 6/2, 40127 Bologna, Italy; 3 Department of Physics and Astronomy, University of Sussex, Brighton, BN1 9QH UK; 4 Alma Mater Research Center on Applied Mathematics - AM2, via saragozza 8, 40123 Bologna, Italy

## Abstract

The formalism of the horizon quantum mechanics is applied to electrically neutral and spherically symmetric black hole geometries emerging from coherent quantum states of gravity to compute the probability that the matter source is inside the horizon. We find that quantum corrections to the classical horizon radius become significant if the matter core has a size comparable to the Compton length of the constituents, and the system is indeed a black hole with probability very close to one unless the core radius is close to the (classical) gravitational radius.

## Introduction

Coherent quantum states can be employed to describe the static and spherically symmetric Schwarzschild black holes as emergent (semi)classical geometries [1].^1^ Such a construction can be straightforwardly extended to the Reissner–Nordström black holes [6, 7], and the semiclassical approximation for rotating geometries can also be obtained from spherically symmetric cases [8–10]. It is important to remark that this approach implies the removal of the central singularities by the presence of a quantum matter core that could, therefore, lead to phenomenological signatures of the kinds analysed in Ref. [11].

The presence of horizons in the above approach can only be established from semiclassical arguments, that is by considering the quantum-corrected metric

1.1 $$\begin{aligned} {\textrm{d}}s^2 = -f(r)\,{\textrm{d}}t^2 + h(r)\,{\textrm{d}}r^2 + r^2\,{\textrm{d}}\Omega ^2 \ , \end{aligned}$$

where $${\textrm{d}}\Omega ^2={\textrm{d}}\theta ^2+\sin ^2\theta \,{\textrm{d}}\phi ^2$$ and

1.2 $$\begin{aligned} f = h^{-1} = 1+2\,V_{\textrm{q}}(r) \ . \end{aligned}$$

In the above, the function $V_{\textrm{q}}=\left\langle V\right| \hat{V}(r)\left| V\right\rangle$ is the expectation value of the relevant metric field on the coherent quantum state $\left| V\right\rangle$. The locations of horizons are then given by solutions $r=r_{\textrm{H}}$ of the classical equation $f(r)=0$. We remark that the Kerr–Schild form (1.1) [12] in which the metric has components $f=h^{-1}$ was chosen because it includes all known spherically symmetric black holes in general relativity. However, one could also consider recovering the semiclassical geometry from a more general coherent quantum state $\left| f,h\right\rangle$ such that $f=\left\langle f,h\right| \hat{f}\left| f,h\right\rangle$, $h=\left\langle f,h\right| \hat{h}\left| f,h\right\rangle$, and $fh\ne 1$, thus extending the approach to the most general form of spherically symmetric geometries in the Abreu–Nielsen–Visser gauge (see [13]). Such a generalisation is left for future developments.

The horizon quantum mechanics was introduced in Refs. [14, 15] (see also Ref. [16] for a review) to compute the probability of the presence of horizons associated with static and spherically symmetric matter sources in a given quantum state $\left| \psi _{\textrm{S}}\right\rangle$. We recall that the Einstein field equations for a source of energy density $\rho =\rho (r)$ imply that^2^

1.3 $$\begin{aligned} f = 1-\frac{2\,G_{\textrm{N}}\,m(r)}{r} \ , \end{aligned}$$

where the Misner–Sharp–Hernandez mass function [17, 18] is given by

1.4 $$m(r) = 4\pi \int_{0}^{r} \rho \left( x \right)x^{2} {\text{d}}x.$$

An horizon then exists if there are values of $r=r_{\textrm{H}}$ such that $2\,G_{\textrm{N}}\,m(r_{\textrm{H}})=r_{\textrm{H}}$. A quantum mechanical description is obtained by replacing the classical energy density with the energy decomposition of the source wavefunction,

1.5 $$\left| {\psi _{{\text{S}}} } \right\rangle = \sum\limits_{E} {\mkern 1mu} C\left( E \right)\left| {\psi _{E} } \right\rangle,$$

where the sum represents the spectral decomposition in Hamiltonian eigenmodes,

1.6 $$\begin{aligned} \hat{H}\left| \psi _E\right\rangle = E\left| \psi _E\right\rangle \ , \end{aligned}$$

and *H* will depend on the model we wish to consider. Upon expressing *E* in terms of the gravitational Schwarzschild radius,^3^$E={r_{\textrm{H}}}/{2\,G_{\textrm{N}}}$, we obtain the horizon wavefunction

1.7 $$\begin{aligned} \psi _{\textrm{H}}(r_{\textrm{H}}) \equiv \langle \, r_{\textrm{H}} \mid \psi _{\textrm{H}}\,\rangle = \mathcal {N}_{\textrm{H}} \sum _{E={r_{\textrm{H}}}/{2\,G_{\textrm{N}}}}\,C(E) \ , \end{aligned}$$

whose normalisation $\mathcal {N}_{\textrm{H}}$ is fixed in the Schrödinger scalar product

1.8 $$\begin{aligned} \langle \, \psi _{\textrm{H}} \mid \phi _{\textrm{H}}\,\rangle = 4\,\pi \int _0^\infty \psi _{\textrm{H}}^*(r_{\textrm{H}})\,\phi _{\textrm{H}}(r_{\textrm{H}})\,r_{\textrm{H}}^2\,{\textrm{d}}r_{\textrm{H}}\ . \end{aligned}$$

The normalised wavefunction yields the probability density for the values of the gravitational radius $r_{\textrm{H}}$ associated with the source in the quantum state $\left| \psi _{\textrm{S}}\right\rangle$, namely

1.9 $$\begin{aligned} \mathcal P_{\textrm{H}}(r_{\textrm{H}}) = 4\,\pi \,r_{\textrm{H}}^2\,|\psi _{\textrm{H}}(r_{\textrm{H}})|^2 \ . \end{aligned}$$

Moreover, the probability density that the source lies inside its own gravitational radius will be given by

1.10 $$\begin{aligned} \mathcal {P}_<(r_{\textrm{H}}) = P_{\textrm{S}}(r_{\textrm{H}})\,\mathcal {P}_{\textrm{H}}(r_{\textrm{H}}) \ , \end{aligned}$$

where

1.11 $$\begin{aligned} P_{\textrm{S}}(r_{\textrm{H}}) = 4\,\pi \,\int _0^{r_{\textrm{H}}} |\psi _{\textrm{S}}(r)|^2\,r^2\,{\textrm{d}}r \end{aligned}$$

is the probability that the source is found inside a sphere of radius $r=r_{\textrm{H}}$. Finally, the probability that the object described by the state $\left| \psi _{\textrm{S}}\right\rangle$ is a black hole will be obtained by integrating Eq. (1.10) over all possible values of the gravitational radius, namely

1.12 $$\begin{aligned} P_{\textrm{BH}} = \int _0^\infty {\mathcal P}_<(r_{\textrm{H}})\,{\textrm{d}}r_{\textrm{H}}\ . \end{aligned}$$

It appears natural to apply the horizon quantum mechanics to black hole geometries described by coherent states and to verify under which conditions there exists a horizon with probability close to one. For this purpose, we will first reconstruct the state $\left| \psi _{\textrm{S}}\right\rangle$ from the effective energy density associated with the quantum-corrected geometry (1.2) in Sect. 2; using that result, we will obtain the horizon wavefunction in Sect. 3; final remarks are given in Sect. 4.

## Coherent quantum states for Schwarzschild geometry

A metric of the form in Eq. (1.1) can be conveniently described as the mean field of the coherent state of a (canonically normalised) free massless scalar field $\sqrt{G_{\textrm{N}}}\,\Phi =(f-1)/2=V$ (see Ref. [1] for all the details). From the Klein–Gordon equation

2.1 $$\begin{aligned} \left[ -\frac{\partial ^2}{\partial t^2} + \frac{1}{r^2}\,\frac{\partial }{\partial r} \left( r^2\,\frac{\partial }{\partial r}\right) \right] \Phi (t,r) = 0 \ , \end{aligned}$$

we obtain the (positive frequency) eigenfunctions

2.2 $$\begin{aligned} u_{k} = {e^{-i \, k \, t}}\,j_0 (k \, r) \ , \end{aligned}$$

where $j_{0}= \sin (k\,r) / k \, r$ with $k>0$ are spherical Bessel functions, which allow us to write the field operator as

2.3 $$\begin{aligned} \hat{\Phi }= \int \limits _0^{\infty } \frac{ k^2 \,{\textrm{d}}k}{2\, \pi ^2} \,\sqrt{\frac{\hbar }{2 \, k}} \left[ u_{k}\, \hat{a}(k) + u_{k }^{*}\, \hat{a}^{\dagger }(k) \right] \end{aligned}$$

and its conjugate momentum as

2.4 $$\begin{aligned} \hat{\Pi }= i\int \limits _0^{\infty } \frac{ k^2 \,{\textrm{d}}k}{2\, \pi ^2} \,\sqrt{\frac{\hbar \, k}{2 }} \left[ u_{k}\, \hat{a}(k) - u_{k }^{*}\, \hat{a}^{\dagger }(k) \right] \ \end{aligned}$$

where $\hat{a}$ and $\hat{a}^{\dagger }$ are the usual annihilation and creation operators.

In particular, we are interested in a coherent state

2.5 $$\begin{aligned} \left| V_M\right\rangle = e^{-N_M/2}\, \exp \!\left\{ \int _0^\infty \frac{k^2\,{\textrm{d}}k}{2\,\pi ^2}\,g_k\,\hat{a}^{\dagger }(k) \right\} \left| 0\right\rangle \ , \end{aligned}$$

which effectively reproduces (as closely as possible) the Schwarzschild geometry, that is

2.6 $$\begin{aligned} \sqrt{G_{\textrm{N}}} \left\langle V_M\right| \hat{\Phi }(t, r ) \left| V_M\right\rangle \simeq V_M(r) = -\frac{2\,G_{\textrm{N}}\,M}{r} \ . \end{aligned}$$

From

2.7 $$\begin{aligned} \left\langle V_M\right| \hat{\Phi }\left| V_M\right\rangle\!\!=\!\! & \int \limits _0^{\infty } \frac{k^2\, {\textrm{d}}k}{2\,\pi ^2}\, \sqrt{\frac{2\,\ell _{\textrm{p}}\,m_{\textrm{p}}}{k}}\, g_{k} \, \cos (k\,t-\gamma _k)\, j_0(k \,r)\, \ , \end{aligned}$$

we impose $\gamma _k= k\, t$ for staticity, and the coefficients $g_{k}$ can be determined by expanding the metric function $V_M=V_M(r)$ on the spatial part of the normal modes (2.2), to obtain

2.8 $$\begin{aligned} g_{k} = -\frac{4\,\pi \,M}{\sqrt{2\,k^3}\,m_{\textrm{p}}} \ . \end{aligned}$$

However, the corresponding normalisation factor

2.9 $$\begin{aligned} N_M = 4\,\frac{M^2}{m_{\textrm{p}}^2} \int \limits _0^{\infty } \frac{{\textrm{d}}k}{k} \end{aligned}$$

diverges both in the infrared and in the ultraviolet. The infrared divergence can be eliminated by embedding the geometry in a universe of finite Hubble radius $r=R_\infty$, whereas the ultraviolet divergence could be removed by assuming the existence of a matter core of finite size $r=R_{\textrm{s}}$.

For the present work, it is convenient to regularise the ultraviolet divergence by replacing the coefficients in Eq. (2.8) with

2.10 $$\begin{aligned} g_{k} = -\frac{4\, \pi \, M\,e^{-\frac{k^2 \, R_{\textrm{s}}^2}{4}}}{\sqrt{2 \, k^3}\,m_{\textrm{p}}} \ , \end{aligned}$$

which yields the total occupation number

2.11 $$\begin{aligned} N_{M} = &\, 4\frac{{M^{2} }}{{m_{p}^{2} }}\int\limits_{{R_{\infty }^{{ - 1}} }}^{\infty } {\frac{{dk}}{k}} e^{{ - \frac{{k^{2} {\kern 1pt} R_{s}^{2} }}{2}}} \\ = &\, 2\frac{{M^{2} }}{{m_{p}^{2} }}\Gamma \left( {0,\frac{{R_{s}^{2} }}{{2R_{\infty }^{2} }}} \right) \\ \simeq &\, 4\frac{{M^{2} }}{{m_{p}^{2} }}\ln \left( {\frac{{R_{\infty } }}{{R_{s} }}} \right) \\ \end{aligned}$$

where $\Gamma =\Gamma (a,x)$ is the incomplete gamma function, and we assumed $R_{\textrm{s}}\ll R_\infty$. The coherent state $\left| V_M\right\rangle$ so defined corresponds to a quantum-corrected metric function

2.12 $$\begin{aligned} V_{\textrm{q}M} = \sqrt{G_{\textrm{N}}} \left\langle V_M\right| \hat{\Phi }\left| V_M\right\rangle = -\frac{G_{\textrm{N}}\,M}{r} \, \textrm{erf} \!\left( \frac{r}{R_{\textrm{s}}} \right) \ , \end{aligned}$$

where $\textrm{erf}$ denotes the error function, and we let $R^{-1}_{\infty } \rightarrow 0$.

### Effective energy density

From the definition of the mass function in Eq. (1.4) and

2.13 $$\begin{aligned} 1+2\,V_{\textrm{q}M} = 1-\frac{2\,G_{\textrm{N}}\,m}{r} \ , \end{aligned}$$

we easily obtain

2.14 $$\begin{aligned} \rho (r) = -\frac{V_{\textrm{q}M}}{4\,\pi \,G_{\textrm{N}}\,r^2} \left( 1+r\,\frac{V_{\textrm{q}M}'}{V_{\textrm{q}M}}\right) \ . \end{aligned}$$

We next note that the quantum-corrected potential (2.12) is of the form

2.15 $$\begin{aligned} V_{\textrm{q}M} = V_M(r)\,v(r) \ , \end{aligned}$$

where the function *v* has the asymptotic behaviours

2.16 $$\begin{aligned} v(r\rightarrow 0)\rightarrow 0 \qquad \textrm{and} \qquad v(r\gg R_{\textrm{s}})\rightarrow 1 \ . \end{aligned}$$

The effective energy density, therefore, reads

2.17 $$\begin{aligned} \rho = \frac{M\,v'}{4\,\pi \,r^2} \ , \end{aligned}$$

so that Eq. (2.16) implies

2.18 $$\begin{aligned} m(r\rightarrow \infty ) = M \int _0^\infty v'(x)\,{\textrm{d}}x = M \ , \end{aligned}$$

as expected.

In particular, we have $v=\textrm{erf}({r}/{R_{\textrm{s}}})$ and

2.19 $$\begin{aligned} \rho = \frac{M\, e^{-\frac{r ^2 }{ R_{\textrm{s}}^2}}}{ {2} \, \pi ^{\frac{3}{2}} \, R_{\textrm{s}} \, r^2} \ , \end{aligned}$$

which is the same result one would obtain from the Einstein field equations $G^\mu _{\ \nu }=8\,\pi \,G_{\textrm{N}}\,T^\mu _{\ \nu }$, where $G^\mu _{\ \nu }$ is the Einstein tensor for the quantum-corrected metric from Eq. (2.12).

## Horizon quantum mechanics

We are interested in a matter source with energy density (2.19) made of a very large number *N* of particles. For simplicity, we assume that all particles are identical and have a mass $\mu = M / N$.

The (normalised) wavefunction of each particle in position space can be estimated as

3.1 $$\begin{aligned} \psi _{\textrm{S }}(r _{i} ) \propto \rho ^{1/2} \propto \frac{e^{-\frac{r _{i} ^2 }{2 \, R_{\textrm{s}}^2}} }{\sqrt{2} \, \pi ^{\frac{3}{4}} \, R_{\textrm{s}}^{\frac{1}{2} } \, r _{i}} \ , \end{aligned}$$

where $i=1,\ldots ,N$. In momentum space, we then have

3.2 $$\begin{aligned} \psi _{\textrm{S}}(k_{i}) = \frac{2 \, \pi ^{\frac{3}{4}} \, R_{\textrm{s}}^{\frac{1}{2} } }{k_{i}} \, \textrm{erfi} \!\left( \frac{ k_{i} \, R_{\textrm{s}}}{\sqrt{2}} \right) \, e^{- \frac{ k_{i}^2 \, R_{\textrm{s}}^2 }{ {2} } } \ , \end{aligned}$$

where $\textrm{erfi}$ is the imaginary error function. Notice that the wavefunction (3.2) peaks around $k = R_\infty ^{-1}$, and the imaginary error function can be approximated for $k_i\,R_{\textrm{s}}\ll 1$ as

3.3 $$\begin{aligned} \textrm{erfi}\!\left( \frac{ k_{i} \, R_{\textrm{s}}}{\sqrt{2}} \right) \simeq \sqrt{\frac{2}{\pi }} \, k_{i} \, R_{\textrm{s}} \ . \end{aligned}$$

Each particle can, therefore, be assumed in a state described by^4^

3.4 $$\begin{aligned} \left| \psi _{\textrm{s}}^{(i)}\right\rangle \simeq \mathcal {N}_k \int \limits _{R_\infty ^{-1}}^{\infty } {{\textrm{d}}k_i}\, e^{- \frac{ k_{i}^2 \, R_{\textrm{s}}^2 }{ {2} } } \,\left| k_i\right\rangle \ , \end{aligned}$$

where $\mathcal {N}_k$ is a suitable normalisation factor.

The dynamics of each particle is determined by a Hamiltonian $H_i$ with spectrum

3.5 $$\begin{aligned} \hat{H}_i \left| E_i\right\rangle = E_i \left| E_i\right\rangle \ , \end{aligned}$$

where

3.6 $$\begin{aligned} E_{i}^2 = \mu ^2+ \hbar ^2 \, k_{i}^2 \ . \end{aligned}$$

Thus, we can rewrite the state (3.4) of each particle as

3.7 $$\left| {\psi _{s}^{{(i)}} } \right\rangle \simeq {\mathcal{N}}_{E} \int\limits_{\mu }^{\infty } d E_{i} e^{{ - \frac{{\left( {E_{i}^{2} - \mu ^{2} } \right)R_{s}^{2} }}{{2m_{p}^{2} \ell _{p}^{2} }}}} \left| {E_{i} } \right\rangle$$

where $\mathcal {N}_E$ is also a normalisation factor.

The total wavefunction of the source will be given by the symmetrised product of *N* such states,

3.8 $$\begin{aligned} \left| \psi _{N}\right\rangle \simeq \frac{ 1 }{N!} \, \sum _{ \{\sigma _i \}}^{N} \, \left[ \bigotimes _{i=1}^{N} \left| \psi _{\textrm{s}}^{(i)}\right\rangle \right] \ , \end{aligned}$$

where the sum is over all the permutations $\{\sigma _i \}$ of the *N* states.

### Source spectral decomposition

The above $\left| \psi _{N}\right\rangle$ can be decomposed into eigenstates $\left| E\right\rangle$ of the total Hamiltonian^5^

3.9 $$\begin{aligned} H = \sum _{i=1}^N H_i = \sum _{i=1}^N \left( \mu ^2+ \hbar ^2 \, k_{i}^2 \right) ^{1/2} \ . \end{aligned}$$

The details of the (approximate analytical) calculation are shown in Appendix A, where we find that $C(E)\equiv \langle E\left| \psi _{N}\right\rangle \simeq 0$, for $E<M$, and

3.10 $$\begin{aligned} C(E) \simeq \mathcal {N}_c \left( \frac{E-M}{m_{\textrm{p}}}\right) ^{M/\mu } e^{-\frac{R_{\textrm{s}}^2\,\mu \,(E-M)}{\ell _{\textrm{p}}^2\,m_{\textrm{p}}^2}} \ , \end{aligned}$$

for $E>M$, with the normalisation constant $\mathcal {N}_c=\mathcal {N}_+$ given in Eq. (A.19). This result means that we can describe the quantum state $\left| \psi _{N}\right\rangle$ of our *N*-particle system by means of the effective one-particle state

3.11 $$\begin{aligned} \left| \Psi _{\textrm{S}}\right\rangle \simeq \mathcal {N}_{\textrm{S}} \int \limits _{M}^{\infty } {\textrm{d}}E \, \left( \frac{E-M}{m_{\textrm{p}}}\right) ^{M/\mu } e^{-\frac{R_{\textrm{s}}^2\,\mu \,(E-M)}{\ell _{\textrm{p}}^2\,m_{\textrm{p}}^2}}\,\left| E\right\rangle \ , \end{aligned}$$

with $E^2 = M^2 + \hbar ^2\, k^2$ and $\mathcal {N}_{\textrm{S}}$ is a normalisation constant. For example, the expectation value of the total energy can be approximated with its upper bound computed in Eq. (A.27) and reads

3.12 $$\begin{aligned} \langle \, \hat{H}\,\rangle \simeq M \left( 1 + \frac{m_{\textrm{p}}^2\,\ell _{\textrm{p}}^2}{\mu ^2\,R_{\textrm{s}}^2} \right) = M \left( 1 + \frac{\lambda _\mu ^2}{R_{\textrm{s}}^2} \right) \ , \end{aligned}$$

where $\lambda _\mu$ is the Compton length of the constituent particles of mass $\mu$. We notice that the relative correction becomes negligibly small for $R_{\textrm{s}}\gg \lambda _\mu$ and diverges for $R_{\textrm{s}}\rightarrow 0$. This is another indication that no well-defined coherent state exists for a pure Schwarzschild geometry [1].

### Horizon wavefunction

We can now obtain the horizon wavefunction from the effective single-particle wavefunction (3.11) by setting $r_{\textrm{H}} = 2\,G_{\textrm{N}}\, E$ and defining $\left| r_{\textrm{H}}\right\rangle \propto \left| 2\,\ell _{\textrm{p}}\, E / m_{\textrm{p}}\right\rangle$. This yields $\Psi _{\textrm{H}}(r_{\textrm{H}})\simeq 0$, for $r_{\textrm{H}}<R_{\textrm{H}}=2\,G_{\textrm{N}}\,M$, and

3.13 $$\begin{aligned} \Psi _{\textrm{H}}(r_{\textrm{H}}) \simeq \mathcal {N}_{\textrm{H}} \left( \frac{r_{\textrm{H}}-R_{\textrm{H}}}{\ell _{\textrm{p}}}\right) ^{\frac{m_{\textrm{p}}\,R_{\textrm{H}}}{2\,\mu \,\ell _{\textrm{p}}}} e^{-\frac{\mu \left( r_{\textrm{H}} - R_{\textrm{H}} \right) R_{\textrm{s}}^2}{2\,m_{\textrm{p}}\,\ell _{\textrm{p}}^3}} \ , \end{aligned}$$

for $r_{\textrm{H}}\ge R_{\textrm{H}}$, where the normalisation constant $\mathcal {N}_{\textrm{H}}$ is given in Eq. (B.2).

The expectation value of the gravitational radius is computed in Eq. (B.3) and can be written as follows:

3.14 $$\begin{aligned} \langle \, \hat{r}_{\textrm{H}}\,\rangle \simeq R_{\textrm{H}}\left( 1 + \frac{\lambda _\mu ^2}{R_{\textrm{s}}^2} \right) \ , \end{aligned}$$

which is in perfect agreement with the expression of the energy given in Eq. (3.12). It is again noteworthy that $\langle \, \hat{r}_{\textrm{H}}\,\rangle >R_{\textrm{H}}$, although the correction with respect to the classical expression is negligible for an astrophysical black hole unless the core is of a size comparable to the Compton length $\lambda _\mu$. It is also important to recall that $\langle \, \hat{r}_{\textrm{H}}\,\rangle$ is the horizon radius only if the core is sufficiently smaller, as we will determine next.

**Fig. 1:**
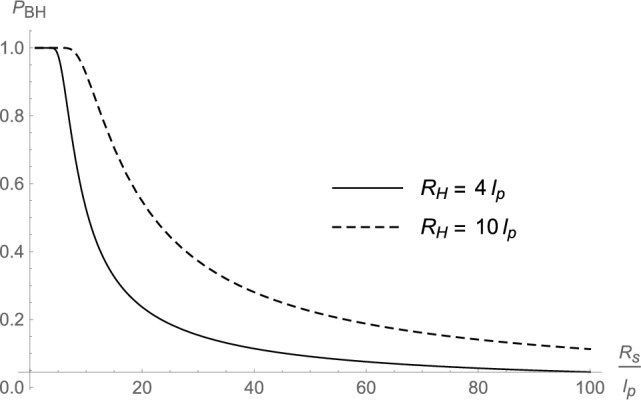
Probability that the coherent state is a black hole as a function of $R_{\textrm{s}}$ for different values of $R_{\textrm{H}}$ (and same value of $\mu =0.2\,m_{\textrm{p}}$)

**Fig. 2:**
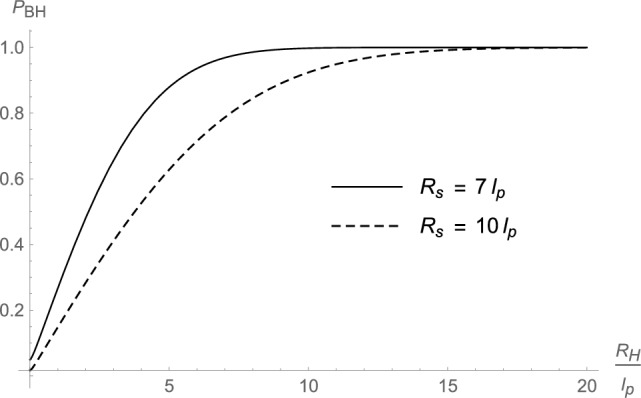
Probability that the coherent state is a black hole as a function of $R_{\textrm{H}}$ for different values of ${R_{\textrm{s}}}$ (and same value of $\mu =0.2\,m_{\textrm{p}}$)

**Fig. 3:**
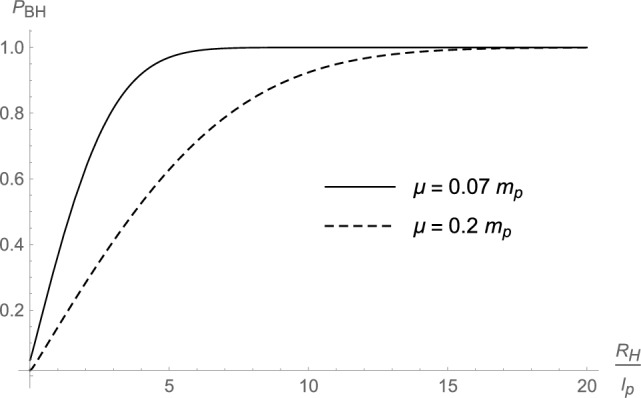
Probability that the coherent state is a black hole as a function of $R_{\textrm{H}}$ for different values of ${\mu }$ (and same value of $R_{\textrm{s}}=10\,\ell _{\textrm{p}}$)

By means of the effective single-particle wavefunction (3.11) and the horizon wavefunction (3.13), we can numerically compute the probability $P_{\textrm{BH}}$ defined in Eq. (1.12) that the system lies inside its own gravitational radius and is a black hole, as reviewed in Sect. 1. More details of the calculation are given in Appendix B, where we show that the final expression of $P_{\textrm{BH}}$ can only be estimated numerically. Some cases are displayed in Figs. 1, 2, and 3, with values of $R_{\textrm{H}}$, $R_{\textrm{s}}$, and $\mu$ chosen for clarity, albeit they fall far from any astrophysical regimes. From those graphs, it appears that the probability increases for decreasing size $R_{\textrm{s}}$ of the core and for increasing (decreasing) mass *M* ($\mu$) (equivalent to increasing $R_{\textrm{H}}=2\,G_{\textrm{N}}\,M$ or the number $N=M/\mu$ of matter particles). For example, a core of size $R_{\textrm{s}}=10\,\ell _{\textrm{p}}$ can be a black hole of radius $R_{\textrm{H}}=10\,\ell _{\textrm{p}}$ with probability $P_{\textrm{BH}}\gtrsim 0.9$ but this probability drops to $P_{\textrm{BH}}\lesssim 0.5$ if $R_{\textrm{H}}=4\,\ell _{\textrm{p}}$. This result is in qualitative agreement with the expectation (3.14) for very massive black holes with cores larger than $\lambda _\mu$, but smaller than the classical gravitational radius $R_{\textrm{H}}$.

## Conclusions and outlook

We have here employed the formalism of the horizon quantum mechanics [14] in order to verify that coherent state black hole geometries of the Schwarzschild type sourced by a core of large mass *M* and with a size $R_{\textrm{s}}$ larger than Planckian are very likely to display an outer horizon and be black holes in the usual sense. For that purpose, we needed to find an explicit description of the (electrically neutral and spherically symmetric) matter core in terms of a many-particle state that was then expressed as a superposition of total energy eigenstates.

Our analysis supports the conclusion that the system is indeed a black hole of mass $M\gg m_{\textrm{p}}$ if its core made of particles of mass $\mu$ has a size $R_{\textrm{s}}\gtrsim \lambda _\mu \gg \ell _{\textrm{p}}$ but (sufficiently) smaller than the classical gravitational radius $R_{\textrm{H}}=2\,G_{\textrm{N}}\,M$. It would be interesting to generalise the above analysis to include electric charge and rotation. Whereas the former case should be rather straightforward, including rotation is going to be much more problematic since it will require extending the horizon quantum mechanics beyond the perturbative regime considered in Ref. [20].

Clearly, the emerging picture is that the (location of the) horizon in quantum physics is fuzzy and, at least for sufficiently large matter cores, one would have “quasi” black hole geometries. In such a picture, the late stage of binary black hole mergers would open a window into quantum features of the gravitational collapse that might affect the emission of gravitational waves at the very peak, or during the ring-down phase, for instance by affecting the black hole Love numbers [21] and possible echos [22, 23]. Of course, quantitative predictions for such effects would require specific analysis that go beyond the scope of the present work.

## Data Availability

No datasets were generated or analysed during the current study.

## Appendix A: Spectral decomposition and total energy

Here, we show how the total wavefunction (3.8), that is

A.1 $$\begin{aligned} \left| \psi _{N}\right\rangle \simeq \frac{ 1 }{N!} \, \sum _{ \{\sigma _i \}}^{N} \, \left[ \bigotimes _{i=1}^{N} \mathcal {N}_E \int \limits _{\mu }^{\infty } {\textrm{d}}E_i \, e^{- \frac{\left( E_i^2 - \mu ^2 \right) \, R_{s}^2}{2 \,m_{\textrm{p}}^2\, \ell _{\textrm{p}}^2}} \, \left| E_i\right\rangle \right] \ , \end{aligned}$$

can be decomposed in terms of the total energy eigenstates $\left| E\right\rangle$ by computing the spectral coefficients $C(E) \equiv \langle \, E \mid \psi _{N}\,\rangle$. From Eq. (A.1), we first find

A.2 $$\begin{aligned} C(E) = & \frac{1}{{N!}}\langle E|\sum\limits_{{\{ \sigma _{i} \} }}^{N} {\left[ { \otimes _{{i = 1}}^{N} {\mathcal{N}}_{E} \int\limits_{\mu }^{\infty } {\text{d}} E_{i} {\mkern 1mu} e^{{ - \frac{{\left( {E_{i}^{2} - \mu ^{2} } \right){\kern 1pt} R_{s}^{2} }}{{2{\kern 1pt} m_{{\text{p}}}^{2} {\kern 1pt} \ell _{{\text{p}}}^{2} }}}} {\mkern 1mu} \left| {E_{i} } \right\rangle } \right]} \\ = & \frac{{{\mathcal{N}}_{E}^{N} }}{{N!}}\int\limits_{\mu }^{\infty } {\text{d}} E_{1} \cdots \int\limits_{\mu }^{\infty } {\text{d}} E_{N} \left[ {\prod\limits_{{i = 1}}^{N} {e^{{ - \frac{{\left( {E_{i}^{2} - \mu ^{2} } \right){\kern 1pt} R_{s}^{2} }}{{2{\kern 1pt} m_{{\text{p}}}^{2} {\kern 1pt} \ell _{{\text{p}}}^{2} }}}} } } \right]\delta \left( {E - \sum\limits_{{i = 1}}^{N} {E_{i} } } \right) \\ \end{aligned}$$

Since $\sum _{i=1}^N E_i\ge N\,\mu =M$, it follows that $C(E<M)=0$. For $E\ge M$, we can use $E_N=\sum _{i=1}^{N-1} E_i$ and write

A.3 $$C(E) \propto \int\limits_{\mu }^{\infty } {\text{d}} E_{1} \cdots \int\limits_{\mu }^{\infty } {\text{d}} E_{{N - 1}} {\text{exp}}\left\{ { - \sum\limits_{{i = 1}}^{{N - 1}} {\frac{{\left( {E_{i}^{2} - \mu ^{2} } \right)R_{s}^{2} }}{{2{\mkern 1mu} m_{{\text{p}}}^{2} {\mkern 1mu} \ell _{{\text{p}}}^{2} }}} - \frac{{\left[ {\left( {E - \sum\limits_{{i = 1}}^{{N - 1}} {\mkern 1mu} E_{i} } \right)^{2} - \mu ^{2} } \right]R_{s}^{2} }}{{2{\mkern 1mu} m_{{\text{p}}}^{2} {\mkern 1mu} \ell _{{\text{p}}}^{2} }}} \right\}$$

It is now convenient to define the function

A.4 $$\begin{aligned} F(E,E_{i} ) \equiv & \sum\limits_{{i = 1}}^{{N - 1}} {\left( {E_{i}^{2} - \mu ^{2} } \right)} + \left( {E - \sum\limits_{{i = 1}}^{{N - 1}} {E_{i} } } \right)^{2} \\ & - \mu ^{2} = \sum\limits_{{i = 1}}^{{N - 1}} {\left( {{\mathcal{E}}_{i} + \mu } \right)^{2} - N\mu ^{2} } + \left[ {E - \sum\limits_{{i = 1}}^{{N - 1}} {{\mathcal{E}}_{i} - (N - 1)\mu } } \right]^{2} \\ \end{aligned}$$

where $\mathcal {E}_i = E_{i} -\mu$. By recalling that $M = N \, \mu$, we then obtain

A.5 $$\begin{aligned} F(E,E_{i} ) = & \sum\limits_{{i = 1}}^{{N - 1}} {\left( {{\mathcal{E}}_{i} + \mu } \right)^{2} } - \mu M + \left[ {\left( {E - M} \right) - \sum\limits_{{i = 1}}^{{N - 1}} {\mkern 1mu} {\mathcal{E}}_{i} + \mu } \right]^{2} \\ = & \left( {E - M} \right)^{2} + \left( {\sum\limits_{{i = 1}}^{{N - 1}} {{\mathcal{E}}_{i} } } \right)^{2} + \mu ^{2} - 2\left( {E - M} \right)\sum\limits_{{i = 1}}^{{N - 1}} {{\mathcal{E}}_{i} + 2\mu \left( {E - M} \right) - 2\mu } \sum\limits_{{i = 1}}^{{N - 1}} {{\mathcal{E}}_{i} } \\ & + \sum\limits_{{i = 1}}^{{N - 1}} {{\mathcal{E}}_{i}^{2} } + 2\mu \sum\limits_{{i = 1}}^{{N - 1}} {{\mathcal{E}}_{i} } + (N - 1){\mkern 1mu} \mu ^{2} - \mu M \\ = & \left( {E - M} \right)^{2} + 2\mu \left( {E - M} \right) - 2\left( {E - M} \right)\sum\limits_{{i = 1}}^{{N - 1}} {{\mathcal{E}}_{i} } + \left( {\sum\limits_{{i = 1}}^{{N - 1}} {{\mathcal{E}}_{i} } } \right)^{2} + \sum\limits_{{i = 1}}^{{N - 1}} {{\mathcal{E}}_{i}^{2} } \\ = & \left[ {E - (M - \mu )} \right]^{2} - \mu ^{2} - 2\left( {E - M} \right)\sum\limits_{{i = 1}}^{{N - 1}} {{\mathcal{E}}_{i} } + \left( {\sum\limits_{{i = 1}}^{{N - 1}} {\mkern 1mu} {\mathcal{E}}_{i} } \right)^{2} + \sum\limits_{{i = 1}}^{{N - 1}} {{\mathcal{E}}_{i}^{2} } \\ \end{aligned}$$

Plugging this result into Eq. (A.3) yields

A.6 $$\begin{aligned} C(E) \propto & e^{{ - \frac{{R_{{\text{s}}}^{2} }}{{2\ell _{{\text{p}}}^{2} m_{{\text{p}}}^{2} }}\left\{ {\left[ {E - (M - \mu )} \right]^{2} - \mu ^{2} } \right\}}} \int\limits_{0}^{\infty } {\text{d}} {\mathcal{E}}_{1} \cdots \int\limits_{0}^{\infty } {\text{d}} {\mathcal{E}}_{{N - 1}} \\ & \times \exp \left\{ {\frac{{R_{s}^{2} }}{{2\ell _{{\text{p}}}^{2} m_{{\text{p}}}^{2} }}\left[ {2\left( {E - M} \right)\sum\limits_{{i = 1}}^{{N - 1}} {{\mathcal{E}}_{i} } - \left( {\sum\limits_{{i = 1}}^{{N - 1}} {{\mathcal{E}}_{i} } } \right)^{2} - \sum\limits_{{i = 1}}^{{N - 1}} {{\mathcal{E}}_{i}^{2} } } \right]} \right\} \\ \equiv & e^{{ - \frac{{R_{{\text{s}}}^{2} }}{{2\ell _{{\text{p}}}^{2} m_{{\text{p}}}^{2} }}\left\{ {\left[ {E - (M - \mu )} \right]^{2} - \mu ^{2} } \right\}}} I(E,M) \\ \end{aligned}$$

We next note that, since $\mathcal {E}_i\ge 0$ for $i=1,\ldots ,N-1$, we have

A.7 $$\begin{aligned} 0 \le \sum _{i=1}^{N-1}\mathcal {E}_i^2 \le \left( \sum _{i=1}^{N-1} \, \mathcal {E}_i \right) ^2 \ . \end{aligned}$$

A lower bound $I_-\le I$ is obtained from the upper bound $\sum _i\mathcal E_i^2=(\sum _i\mathcal E_i)^2$ in Eq. (A.7) and is given by

A.8 $$\begin{aligned} I_{ - } = & \int\limits_{0}^{\infty } d {\rm E}_{1} \cdots \int\limits_{0}^{\infty } d {\rm E}_{{N - 1}} \exp \left[ {\frac{{R_{s}^{2} (E - M)}}{{\ell _{p}^{2} m_{p}^{2} }}\sum\limits_{{i = 1}}^{{N - 1}} {{\rm E}_{i} } } \right]\exp \left[ { - \frac{{R_{s}^{2} }}{{\ell _{p}^{2} m_{p}^{2} }}\left( {\sum\limits_{{i = 1}}^{{N - 1}} {{\rm E}_{i} } } \right)^{2} } \right] \\ \propto & \int\limits_{0}^{\infty } {{\rm E}^{{N - 2}} } d{\rm E}\exp \left[ { - \frac{{R_{s}^{2} {\rm E}^{2} }}{{\ell _{p}^{2} m_{p}^{2} }} + \frac{{R_{s}^{2} (E - M){\rm E}}}{{\ell _{p}^{2} m_{p}^{2} }}} \right] \\ = & \frac{1}{2}\left( {\frac{{R_{s} }}{{m_{p} \ell _{p} }}} \right)^{{1 - N}} \Gamma \left( {\frac{{N - 1}}{2}} \right)_{1} F_{1} \left[ {\frac{{N - 1}}{2},\frac{1}{2},\frac{{\left( {E - M} \right)^{2} R_{s}^{2} }}{{4{\mkern 1mu} m_{p}^{2} \ell _{p}^{2} }}} \right] \\ & + \frac{1}{2}\left( {\frac{{R_{s} }}{{m_{p} \ell _{p} }}} \right)^{{1 - N}} \frac{{\left( {E - M} \right)R_{s} }}{{m_{p} \ell _{p} }}\Gamma \left( {\frac{N}{2}} \right){\mkern 1mu} _{1} F_{1} \left[ {\frac{N}{2},\frac{3}{2},\frac{{\left( {E - M} \right)^{2} R_{s}^{2} }}{{4{\mkern 1mu} m_{p}^{2} \ell _{p}^{2} }}} \right], \\ \end{aligned}$$

where $\Gamma$ is the Euler gamma function, and $_{1}F_{1}$ is the Kummer confluent hypergeometric function. An upper bound $I\le I_+$ is likewise obtained from the lower bound $\sum _{i}\mathcal E_i^2=0$ in Eq. (A.7) and is given by

A.9 $$\begin{aligned} I_{ + } = & \int\limits_{0}^{\infty } d \varepsilon _{1} \cdots \int\limits_{0}^{\infty } d \varepsilon _{{N - 1}} \exp \left[ {\frac{{R_{s}^{2} (E - M)}}{{\ell _{p}^{2} m_{p}^{2} }}\sum\limits_{{i = 1}}^{{N - 1}} {\varepsilon _{i} } } \right]\exp \left[ { - \frac{{R_{s}^{2} }}{{2\ell _{p}^{2} m_{p}^{2} }}\left( {\sum\limits_{{i = 1}}^{{N - 1}} {\varepsilon _{i} } } \right)^{2} } \right] \\ \propto & \int\limits_{0}^{\infty } {\varepsilon ^{{N - 2}} } d\varepsilon \exp \left[ { - \frac{{R_{s}^{2} \varepsilon ^{2} }}{{2\ell _{p}^{2} m_{p}^{2} }} + \frac{{R_{s}^{2} (E - M)\varepsilon }}{{\ell _{p}^{2} m_{p}^{2} }}} \right] \\ = & \frac{1}{{2^{{\frac{N}{2} - \frac{3}{2}}} }}\left( {\frac{{R_{s} }}{{m_{p} \ell _{p} }}} \right)^{{1 - N}} \Gamma \left( {\frac{{N - 1}}{2}} \right)_{1} F_{1} \left[ {\frac{{N - 1}}{2},\frac{1}{2},\frac{{\left( {E - M} \right)^{2} R_{s}^{2} }}{{2m_{p}^{2} \ell _{p}^{2} }}} \right] \\ & + \frac{1}{{2^{{\frac{N}{2} - 1}} }}\left( {\frac{{R_{s} }}{{m_{p} \ell _{p} }}} \right)^{{1 - N}} \frac{{\left( {E - M} \right)R_{s} }}{{m_{p} \ell _{p} }}\Gamma \left( {\frac{N}{2}} \right)_{1} F_{1} \left[ {\frac{N}{2},\frac{3}{2},\frac{{\left( {E - M} \right)^{2} R_{s}^{2} }}{{2m_{p}^{2} \ell _{p}^{2} }}} \right] \\ \end{aligned}$$

For $R_{\textrm{s}}\gg \ell _{\textrm{p}}$ and $(E-M)\gtrsim m_{\textrm{p}}$, we can employ the asymptotic behaviour of the Kummer confluent hypergeometric function,

A.10 $$\begin{aligned} _{1}F_{1}\,\left( a , b , x\right) \sim x^{a-b}\,e^{x} \ , \end{aligned}$$

for $x\sim (E-M)^2\,R_{\textrm{s}}^2/\ell _{\textrm{p}}^2\,m_{\textrm{p}}^2\gg 1$, which leads to

A.11 $$\begin{aligned} I_{ - } \simeq & \frac{1}{{2^{{N - 2}} }}\frac{{m_{p} \ell _{p} }}{{R_{s} }}\left( {E - M} \right)^{{N - 2}} e^{{\frac{{\left( {E - M} \right)^{2} R_{s}^{2} }}{{4m_{p}^{2} \ell _{p}^{2} }}}} \left[ {2\Gamma \left( {\frac{{N - 1}}{2}} \right) + \Gamma \left( {\frac{N}{2}} \right)} \right] \\ \simeq & \Gamma \left( {\frac{{N - 1}}{2}} \right)\frac{{m_{p} \ell _{p} }}{{2^{{N - 1}} R_{s} }}\left( {E - M} \right)^{{N - 2}} e^{{\frac{{\left( {E - M} \right)^{2} R_{s}^{2} }}{{4m_{p}^{2} \ell _{p}^{2} }}}} \\ \end{aligned}$$

where we also used $2\,\Gamma \left( (N-1)/2\right) >\Gamma \left( N/2\right)$ for $N\gg 1$ in the last step. Likewise,

A.12 $$\begin{aligned} I_{ + } \simeq & \frac{{m_{p} \ell _{p} }}{{\sqrt 2 R_{s} }}\left( {E - M} \right)^{{N - 2}} e^{{\frac{{\left( {E - M} \right)^{2} R_{s}^{2} }}{{2m_{p}^{2} \ell _{p}^{2} }}}} \left[ {\Gamma \left( {\frac{{N - 1}}{2}} \right) + 2\Gamma \left( {\frac{N}{2}} \right)} \right] \\ \simeq & \sqrt 2 \Gamma \left( {\frac{N}{2}} \right)\frac{{m_{p} \ell _{p} }}{{R_{s} }}\left( {E - M} \right)^{{N - 2}} e^{{\frac{{\left( {E - M} \right)^{2} R_{s}^{2} }}{{2m_{p}^{2} \ell _{p}^{2} }}}} \\ \end{aligned}$$

where we used $2\,\Gamma \!\left( N/2\right) >\Gamma \!\left( (N-1)/2\right)$ for $N\gg 1$.

Therefore, we have

A.13 $$\begin{aligned} \Gamma \! \left( \frac{N-1}{2}\right) \frac{m_{\textrm{p}}\,\ell _{\textrm{p}}}{2^{N - 1}\,R_{\textrm{s}}} \left( E-M\right) ^{N-2} e^{\frac{\left( E-M \right) ^2 R_{\textrm{s}}^2}{ 4 \, m_{\textrm{p}}^2\,\ell _{\textrm{p}}^2} } \lesssim I \lesssim \sqrt{2}\, \Gamma \! \left( \frac{N}{2}\right) \frac{m_{\textrm{p}}\,\ell _{\textrm{p}}}{R_{\textrm{s}}} \left( E-M\right) ^{N-2} e^{\frac{\left( E-M \right) ^2 R_{\textrm{s}}^2}{ 2 \, m_{\textrm{p}}^2\,\ell _{\textrm{p}}^2} } \ . \end{aligned}$$

We might note that the above approximation fails for $0<(E-M)\lesssim m_{\textrm{p}}$ at fixed value of $R_{\textrm{s}}\gtrsim \ell _{\textrm{p}}$, for which we instead find the Taylor expansion

A.14 $$\begin{aligned} I_+ \sim I_- \propto 1 + \mathcal {O}\left( \frac{E-M}{m_{\textrm{p}}}\right) \ . \end{aligned}$$

However, for an astrophysical system of mass $M\gg m_{\textrm{p}}$, this regime can be discarded overall.

By recalling that $N=M/\mu \gg 1$, we finally obtain the bounding functions

A.15 $$\begin{aligned} C_-(E) = \mathcal {N}_- \left( \frac{E-M}{m_{\textrm{p}}}\right) ^{M/\mu } e^{-\frac{R_{\textrm{s}}^2\,\mu \,(E-M)}{\ell _{\textrm{p}}^2\,m_{\textrm{p}}^2}}\, e^{-\frac{R_{\textrm{s}}^2\left( E-M \right) ^2}{ 4 \, m_{\textrm{p}}^2\,\ell _{\textrm{p}}^2} } \end{aligned}$$

and

A.16 $$\begin{aligned} C_+(E)\!\!=\!\! & \mathcal {N}_+ \left( \frac{E-M}{m_{\textrm{p}}}\right) ^{M/\mu } e^{-\frac{R_{\textrm{s}}^2\,\mu \,(E-M)}{\ell _{\textrm{p}}^2\,m_{\textrm{p}}^2}} \ . \end{aligned}$$

The normalisations $\mathcal {N}_{\pm }$ can be obtained from the condition

A.17 $$\begin{aligned} 1 = \int \limits _{M}^{\infty } C^2_\pm (E) \,{\textrm{d}}E \ , \end{aligned}$$

yielding

A.18 $$\begin{aligned} {\mathcal{N}}_{ - }^{{ - 2}} = & \left( {\frac{{\ell _{p} }}{{\sqrt 2 R_{s} }}} \right)^{{\frac{{2M}}{\mu }}} \Gamma \left( {1 + \frac{{2M}}{\mu }} \right)U\left( {1 + \frac{M}{\mu },\frac{3}{2},\frac{{2\mu ^{2} R_{s}^{2} }}{{m_{p}^{2} \ell _{p}^{2} }}} \right) \\ \simeq & \left( {\frac{{\ell _{p} }}{{\sqrt 2 R_{s} }}} \right)^{{\frac{{2M}}{\mu }}} \Gamma \left( {\frac{{2M}}{\mu }} \right)U\left( {\frac{M}{\mu },\frac{3}{2},\frac{{2\mu ^{2} R_{s}^{2} }}{{m_{p}^{2} \ell _{p}^{2} }}} \right) \\ \end{aligned}$$

where $U=U(a,b,x)$ is the Tricomi confluent hypergeometric function, and

A.19 $$\begin{aligned} {\mathcal{N}}_{ + }^{{ - 2}} = & m_{{\text{p}}} \left( {\frac{{m_{{\text{p}}} \ell _{{\text{p}}}^{2} }}{{2\mu R_{{\text{s}}}^{2} }}} \right)^{{1 + \frac{{2M}}{\mu }}} \Gamma \left( {1 + \frac{{2M}}{\mu }} \right) \\ \simeq & m_{{\text{p}}} \left( {\frac{{m_{{\text{p}}} \ell _{{\text{p}}}^{2} }}{{2\mu R_{{\text{s}}}^{2} }}} \right)^{{\frac{{2M}}{\mu }}} \Gamma \left( {\frac{{2M}}{\mu }} \right) \\ \end{aligned}$$

In Sect. 3.1, we use the upper bounding function (A.16) in order to estimate the maximum corrections to the total energy. In fact, the bounds from the spectral coefficients can be used to bound the expectation value of the total energy as

A.20 $$\begin{aligned} M+H_- \lesssim \langle \, \hat{H}\,\rangle \lesssim M+H_+ \ , \end{aligned}$$

where

A.21 $$\begin{aligned} H_{ + } = & \int\limits_{M}^{\infty } {C_{ + }^{2} } (E)EdE - M \\ = & \int\limits_{0}^{\infty } {C_{ + }^{2} } (\varepsilon )\varepsilon d\varepsilon \\ = & \frac{{\ell _{p}^{2} m_{p}^{2} (2M + \mu )}}{{2\mu ^{2} R_{s}^{2} }} \\ \simeq & M\frac{{m_{p}^{2} \ell _{p}^{2} }}{{\mu ^{2} R_{s}^{2} }} \\ \end{aligned}$$

and

A.22 $$\begin{aligned} H_- = \int \limits _{0}^{\infty } C_-^2(\mathcal {E}) \, \mathcal {E}\,{\textrm{d}}\mathcal {E} = \frac{ \ell _{\textrm{p}}^2\,m_{\textrm{p}}^2\,(2\,M+\mu )\, U\!\left( 1+\frac{M}{\mu },\frac{1}{2},\frac{2\,\mu ^2\,R_{\textrm{s}}^2}{m_{\textrm{p}}^2\,\ell _{\textrm{p}}^2}\right) }{2\,\mu ^2\,R_{\textrm{s}}^2\, U\!\left( 1+\frac{M}{\mu },\frac{3}{2},\frac{2\,\mu ^2\,R_{\textrm{s}}^2}{m_{\textrm{p}}^2\,\ell _{\textrm{p}}^2}\right) } \ . \end{aligned}$$

Employing the definition of the Tricomi confluent hypergeometric function,

A.23 $$\begin{aligned} U(a,b,x) = \frac{\Gamma \left( 1-b \right) }{\Gamma \left( a+1-b \right) } \, _{1}F_{1}\,\left( a , b , x\right) + \frac{\Gamma \left( b-1 \right) }{\Gamma \left( a \right) } \, x^{1-b} \, _{1}F_{1}\,\left( a+1-b , 2 - b , x\right) \ , \end{aligned}$$

and the asymptotic behaviour of the Kummer confluent hypergeometric function (A.10), we find

A.24 $$\begin{aligned} U\!\left( 1+\frac{M}{\mu },\frac{1}{2},\frac{2\,\mu ^2\,R_{\textrm{s}}^2}{m_{\textrm{p}}^2\,\ell _{\textrm{p}}^2}\right) \simeq \left[ \frac{1}{\Gamma \!\left( \frac{M}{\mu }+ \frac{3}{2} \right) } -\frac{2}{\Gamma \!\left( \frac{M}{\mu }+1 \right) } \right] \Gamma \!\left( \frac{1}{2} \right) \left( \frac{\sqrt{2 }\,\mu \,R_{\textrm{s}}}{m_{\textrm{p}}\,\ell _{\textrm{p}}} \right) ^{1+2 \, \frac{M}{\mu }} e^{\frac{2\,\mu ^2\,R_{\textrm{s}}^2}{m_{\textrm{p}}^2\,\ell _{\textrm{p}}^2}} \end{aligned}$$

and

A.25 $$\begin{aligned} U\!\left( 1+\frac{M}{\mu },\frac{3}{2},\frac{2\,\mu ^2\,R_{\textrm{s}}^2}{m_{\textrm{p}}^2\,\ell _{\textrm{p}}^2}\right) \simeq \left[ \frac{1}{\Gamma \!\left( \frac{M}{\mu }+ 1 \right) } -\frac{2}{\Gamma \!\left( \frac{M}{\mu }+\frac{1}{2} \right) } \right] \Gamma \left( \frac{1}{2} \right) \left( \frac{\sqrt{2 }\,\mu \,R_{\textrm{s}}}{m_{\textrm{p}}\,\ell _{\textrm{p}}} \right) ^{2 \, \frac{M}{\mu } -1} e^{\frac{2\,\mu ^2\,R_{\textrm{s}}^2}{m_{\textrm{p}}^2\,\ell _{\textrm{p}}^2}} \ , \end{aligned}$$

from which

A.26 $$\begin{aligned} H_{ - } \simeq & (2M + \mu )\frac{{\Gamma \left( {\frac{M}{\mu } + \frac{1}{2}} \right)\left[ {\Gamma \left( {\frac{M}{\mu } + 1} \right) - 2\Gamma \left( {\frac{M}{\mu } + \frac{3}{2}} \right)} \right]}}{{\Gamma \left( {\frac{M}{\mu } + \frac{3}{2}} \right)\left[ {\Gamma \left( {\frac{M}{\mu } + \frac{1}{2}} \right) - 2\Gamma \left( {\frac{M}{\mu } + 1} \right)} \right]}} \\ \simeq & 2\mu \left( {\frac{M}{\mu }} \right)^{{\frac{1}{2}}} \\ \end{aligned}$$

Putting the above bounds together, we obtain

A.27 $$\begin{aligned} M \left[ 1 + 2 \, \left( \frac{\mu }{M} \right) ^{\frac{1}{2}} \right] \lesssim \langle \, \hat{H}\,\rangle \lesssim M \left( 1+ \frac{\lambda _\mu ^2}{R_{\textrm{s}}^2} \right) \ , \end{aligned}$$

which shows that $\langle \, \hat{H}\,\rangle$ cannot be smaller than the classical ADM mass, and the (maximum) relative correction is proportional to the Compton length $\lambda _\mu =\ell _{\textrm{p}}\,m_{\textrm{p}}/\mu$.

## Appendix B: Horizon wavefunction and black hole probability

In Sect. 3.2, we continue to employ the upper bound on the spectral decomposition to obtain the horizon wavefunction in Eq. (3.13) and estimate the maximum possible correction to the gravitational radius and minimum probability $P_{\textrm{BH}}$. Its normalisation is given by

B.1 $$\begin{aligned} 1 = 4\,\pi \int \limits _{R_{\textrm{H}}}^\infty \left| \Psi _{\textrm{H}}(r_{\textrm{H}})\right| ^2 r_{\textrm{H}}^2\, {\textrm{d}}r_{\textrm{H}}= 4 \, \pi \, \mathcal {N}_{\textrm{H}}^2 \int \limits _{0}^\infty \left( \frac{\tilde{r}_{\textrm{H}}}{\ell _{\textrm{p}}}\right) ^{\frac{m_{\textrm{p}}\,R_{\textrm{H}}}{\mu \,\ell _{\textrm{p}}}} e^{-\frac{\mu \,\tilde{r}_{\textrm{H}}\,R_{\textrm{s}}^2}{m_{\textrm{p}}\,\ell _{\textrm{p}}^3}} \, \left( \tilde{r}_{\textrm{H}} + R_{\textrm{H}} \right) ^2\,{\textrm{d}}\tilde{r}_{\textrm{H}} \ , \end{aligned}$$

where $\tilde{r}_{\textrm{H}}=r_{\textrm{H}}-R_{\textrm{H}}$. The above expression yields

B.2 $$\begin{aligned} {\mathcal{N}}_{{\text{H}}}^{{ - 2}} = & \frac{{8\pi \ell _{{\text{p}}}^{9} {\mkern 1mu} m_{{\text{p}}}^{3} }}{{\mu ^{3} R_{{\text{s}}}^{6} }}\left( {\frac{{m_{{\text{p}}} \ell _{{\text{p}}}^{2} }}{{\mu R_{{\text{s}}}^{2} }}} \right)^{{\frac{{m_{{\text{p}}} {\kern 1pt} R_{{\text{H}}} }}{{\mu {\kern 1pt} \ell _{{\text{p}}} }}}} \left[ {\left( {1 + \frac{{m_{{\text{p}}} R_{{\text{H}}} }}{{\mu \ell _{{\text{p}}} }}} \right)\left( {1 + \frac{{m_{{\text{p}}} R_{{\text{H}}} }}{{2\mu \ell _{{\text{p}}} }} + \frac{{\mu R_{{\text{H}}} R_{{\text{s}}}^{2} }}{{m_{{\text{p}}} \ell _{{\text{p}}}^{3} }}} \right) + \frac{{\mu ^{2} R_{{\text{H}}}^{2} R_{{\text{s}}}^{4} }}{{2\ell _{{\text{p}}}^{6} m_{{\text{p}}}^{2} }}} \right] \\ & \times \Gamma \left( {1 + \frac{{m_{{\text{p}}} {\mkern 1mu} R_{{\text{H}}} }}{{\mu \ell _{{\text{p}}} }}} \right) \simeq \frac{{4\pi \ell _{{\text{p}}}^{3} m_{{\text{p}}} R_{{\text{H}}}^{2} }}{{\mu R_{{\text{s}}}^{2} }}\left( {\frac{{m_{{\text{p}}} \ell _{{\text{p}}}^{2} }}{{\mu R_{{\text{s}}}^{2} }}} \right)^{{\frac{{m_{{\text{p}}} {\kern 1pt} R_{{\text{H}}} }}{{\mu {\kern 1pt} \ell _{{\text{p}}} }}}} \Gamma \left( {\frac{{m_{{\text{p}}} {\mkern 1mu} R_{{\text{H}}} }}{{\mu {\mkern 1mu} \ell _{{\text{p}}} }}} \right) \\ \end{aligned}$$

where we again used $R_{\textrm{s}}\sim R_{\textrm{H}}\gg \ell _{\textrm{p}}$ in the last approximation.

The expectation value of the gravitational radius is given by

B.3 $$\begin{aligned} \langle \Psi _{{\text{H}}} |\hat{r}_{{\text{H}}} \left| {\Psi _{{\text{H}}} } \right\rangle = &\, 4\pi \int\limits_{0}^{\infty } {\left| {\Psi _{{\text{H}}} (r_{{\text{H}}} )} \right|^{2} } r_{{\text{H}}}^{3} {\mkern 1mu} {\text{d}}r_{{\text{H}}} \\ = &\, R_{{\text{H}}} + R_{{\text{H}}} {\mkern 1mu} \frac{{m_{{\text{p}}}^{2} \ell _{{\text{p}}}^{2} }}{{\mu ^{2} R_{{\text{s}}}^{2} }}\left( {1 + \frac{{3\ell _{{\text{p}}} \mu }}{{m_{{\text{p}}} R_{{\text{H}}} }}} \right) \\ & - R_{{\text{H}}} \frac{{\left( {1 + \frac{{m_{{\text{p}}} R_{{\text{H}}} }}{{\mu \ell _{{\text{p}}} }}} \right) + \frac{{\mu R_{{\text{H}}} R_{{\text{s}}}^{2} }}{{\ell _{{\text{p}}}^{3} {\mkern 1mu} m_{{\text{p}}} }}}}{{\left( {1 + \frac{{m_{{\text{p}}} R_{{\text{H}}} }}{{\mu \ell _{{\text{p}}} }}} \right)\left( {1 + \frac{{m_{{\text{p}}} R_{{\text{H}}} }}{{2\ell _{{\text{p}}} \mu }}} \right) + \frac{{\mu {\mkern 1mu} R_{{\text{H}}} R_{{\text{s}}}^{2} }}{{\ell _{{\text{p}}}^{3} {\mkern 1mu} m_{{\text{p}}} }}\left( {1 + \frac{{m_{{\text{p}}} R_{{\text{H}}} }}{{\ell _{{\text{p}}} \mu }}} \right) + \frac{{\mu ^{2} R_{{\text{H}}}^{2} R_{{\text{s}}}^{4} }}{{2\ell _{{\text{p}}}^{6} m_{{\text{p}}}^{2} }}}} \\ \simeq & R_{{\text{H}}} \left[ {1 + \frac{{\lambda _{\mu }^{2} }}{{R_{{\text{s}}}^{2} }}\left( {1 - \frac{{2\ell _{{\text{p}}}^{2} }}{{R_{{\text{H}}} \lambda _{\mu } }}} \right)} \right] \simeq _{{\text{H}}} \left( {1 + \frac{{\lambda _{\mu }^{2} }}{{R_{{\text{s}}}^{2} }}} \right) \\ \end{aligned}$$

with $R_{\textrm{H}}=2 \, G_{\textrm{N}}\,M\gg \lambda _\mu$ the classical Schwarzschild radius and $\lambda _\mu \gg \ell _{\textrm{p}}$ the Compton length of the matter costituents.

The probability density (1.9) for the horizon to be located on the sphere of radius $r = r_{\textrm{H}}$ vanishes for $0\le r_{\textrm{H}}<R_{\textrm{H}}$, else is given by

B.4 $$\begin{aligned} \mathcal {P}_{\textrm{H}}( r_{\textrm{H}}) \simeq 4 \, \pi \, \mathcal {N}_{\textrm{H}}^2 \, r_{\textrm{H}}^2 \, \left( \frac{r_{\textrm{H}}-R_{\textrm{H}}}{\ell _{\textrm{p}}}\right) ^{\frac{m_{\textrm{p}}\,R_{\textrm{H}}}{\mu \,\ell _{\textrm{p}}}} e^{-\frac{\mu \left( r_{\textrm{H}} - R_{\textrm{H}} \right) R_{\textrm{s}}^2}{m_{\textrm{p}}\,\ell _{\textrm{p}}^3}} \ . \end{aligned}$$

The probability density (1.10) can be explicitly computed from the wavefunction (3.1) with $r_i=r$ and the horizon probability density (B.4),

B.5 $$\begin{aligned} \mathcal {P}_{<}(r <r_{\textrm{H}}) &= \left( 4 \,\pi \int \limits _{R_{\textrm{H}}}^{r_{\textrm{H}}} \left| \psi _{\textrm{S}} (r) \right| ^2 r^2 \, {\textrm{d}}r \right) \mathcal {P}_{\textrm{H}}(r_{\textrm{H}}) \nonumber \\ &= \textrm{erf} \left( \frac{r_{\textrm{H}}}{R_{\textrm{s}} } \right) \mathcal {P}_{\textrm{H}}(r_{\textrm{H}}) \ , \quad \end{aligned}$$

where $\textrm{erf}$ denotes the error function. The black hole probability (1.12) now reads

B.6 $$\begin{aligned} P_{\textrm{BH}} = \int \limits _{R_{\textrm{H}}}^{\infty } \mathcal {P}_{<}(r<r_{\textrm{H}})\, {\textrm{d}}r_{\textrm{H}}\ , \end{aligned}$$

which, however, can only be computed numerically for specific values of $R_{\textrm{H}}$, $R_{\textrm{s}}$, and $\mu$ (see Figs. 1, 2, and 3).

## Footnotes

1 For studies of their thermodynamics and configurational entropy, see Refs. [2–4] and, for more phenomenological consequences, see Ref. [5].

2 We shall use units with $c=1$, the Newton constant $G_{\textrm{N}}=\ell _{\textrm{p}}/m_{\textrm{p}}$ and the Planck constant $\hbar =\ell _{\textrm{p}}\,m_{\textrm{p}}$, where $\ell _{\textrm{p}}$ and $m_{\textrm{p}}$ are the Planck length and mass, respectively.

3 For the local version of the formalism, see Ref. [19].

4 Given the approximation (3.3), the expression (3.4) “underestimates” the exact wavefunction at large $k\sim R_{\textrm{s}}^{-1}$. The related error can be reduced by decreasing the value of $R_{\textrm{s}}$ with respect to the (unknown) actual size of the core.

5 Notice that we are assuming that the total energy is just the sum of individual particle energies to parallel the expression (1.4) of the classical mass function.
